# hCLE/C14orf166, a cellular protein required for viral replication, is incorporated into influenza virus particles

**DOI:** 10.1038/srep20744

**Published:** 2016-02-11

**Authors:** Ariel Rodriguez-Frandsen, Susana de Lucas, Alicia Pérez-González, Maite Pérez-Cidoncha, Alejandro Roldan-Gomendio, Alejandra Pazo, Laura Marcos-Villar, Sara Landeras-Bueno, Juan Ortín, Amelia Nieto

**Affiliations:** 1 Centro Nacional de Biotecnología, CSIC, Darwin 3, Cantoblanco, 28049 Madrid, Spain; 2 Ciber de Enfermedades Respiratorias, ISCIII, Spain

## Abstract

The influenza A virus polymerase associates with a number of cellular transcription-related factors, including the RNA polymerase II (RNAP II). We previously described that the cellular protein hCLE/C14orf166 interacts with and stimulates influenza virus polymerase as well as RNAP II activities. Here we show that, despite the considerable cellular shut-off observed in infected cells, which includes RNAP II degradation, hCLE protein levels increase throughout infection in a virus replication-dependent manner. Human and avian influenza viruses of various subtypes increase hCLE levels, but other RNA or DNA viruses do not. hCLE colocalises and interacts with viral ribonucleoproteins (vRNP) in the nucleus, as well as in the cytoplasm late in infection. Furthermore, biochemical analysis of purified virus particles and immunoelectron microscopy of infected cells show hCLE in virions, in close association with viral vRNP. These findings indicate that hCLE, a cellular protein important for viral replication, is one of the very few examples of transcription factors that are incorporated into particles of an RNA-containing virus.

Viruses are mandatory intracellular parasites, since they do not contain all the genetic information required to complete their life cycle. The degree of host cell reliance depends on virus coding capacity and genome expression strategy. The influenza A virus (IAV) genome consists of eight negative-sense RNA segments^1^. To enhance use of the available sequence, IAV has evolved different molecular mechanisms to achieve expression of many proteins from a single RNA segment. These mechanisms include alternative splicing of viral mRNAs as well as non-canonical translation strategies such as leaky ribosomal scanning, non-AUG initiation and ribosomal frameshifting^2^. Ten viral proteins have been studied extensively^1^ but five more proteins expressed from the polymerase-encoding RNA segments have recently been identified^3^, which enlarges the number of possible host cell-virus interactions and would increase the degree of viral replication control.

The polymerase subunits (PA, PB1 and PB2) and the nucleoprotein (NP) are responsible for genome expression. These proteins associate to each viral RNA segment to constitute the viral ribonucleoproteins (vRNP)^4^,^5^. Viral transcription involves a cap-stealing mechanism by which 5′-capped oligonucleotides are used as primers and are elongated by the viral polymerase^6^,^7^. As these primers are derived from newly synthesised RNA polymerase II (RNAP II) transcripts, this mechanism implies a functional coupling between viral and cellular transcription machineries. Within the viral polymerase, the PB2 subunit recognises the cap structure^8^,^9^,^10^, while the PA subunit is needed to cleave capped oligonucleotides^11^,^12^ and the PB1 subunit has the catalytic polymerase activity^13^. In accordance with the viral transcription mechanism, a number of cellular transcription-related factors, including RNAP II^14^, are reported to associate with the viral polymerase complex or its subunits. Other factors that interact with the viral polymerase are *Ebp-1* 15, which represses transcription of cell cycle genes regulated by E2F^16^; DDX5^17^, a transcription co-activator that might have a role in cellular transcription initiation^18^; hStaufen1, a protein involved in cellular mRNA transport that might participate in late events of IAV infection^19^; NXP2/MORC3^17^, a nuclear matrix protein involved in influenza virus transcription^20^ and SFPQ/PSF factor^17^, which increases the efficiency of viral mRNA polyadenylation^21^. We previously reported the interaction of human CLE (hCLE) and the chromatin-remodelling factor CHD6 with the PA polymerase subunit^22^. Further characterisation indicated that both hCLE and CHD6 also interact with the viral polymerase complex in infected cells^23^,^24^. Earlier studies described hCLE association with and positive modulation of RNAP II^25^, and a recent report pointed out that hCLE is a shuttling protein that forms nuclear and cytosolic protein complexes with DDX1, HSPC117 and FAM98B proteins^26^.

Although viral and cellular transcriptional machineries need to be coupled, RNAP II is degraded and cellular transcription is inhibited during IAV infection^27^,^28^,^29^,^30^,^31^,^32^. This degradation takes place once viral mRNA synthesis is completed and cellular transcription is thus no longer required^27^. RNAP II degradation reduces host gene expression and might help to diminish the antiviral response. hCLE interacts with the unphosphorylated and phosphorylated forms of RNAP II. Based on this interaction, cellular transcription is strongly dependent on hCLE, since a notable reduction in cellular mRNA synthesis is observed in hCLE-knockdown conditions^24^,^25^. Both hCLE-RNAP II interaction and hCLE control of mRNA synthesis make hCLE an appropriate target for degradation during infection, to prevent the antiviral response. We examined whether hCLE is degraded during IAV infection, and found that in fact its levels increase in infected cells. We observed that, in addition to its nuclear association, hCLE colocalises and interacts with vRNP in cytoplasm late in infection and is incorporated into virions in close association with packaged vRNP.

## Results

### hCLE accumulation increases following influenza virus infection

**Figure 1:**
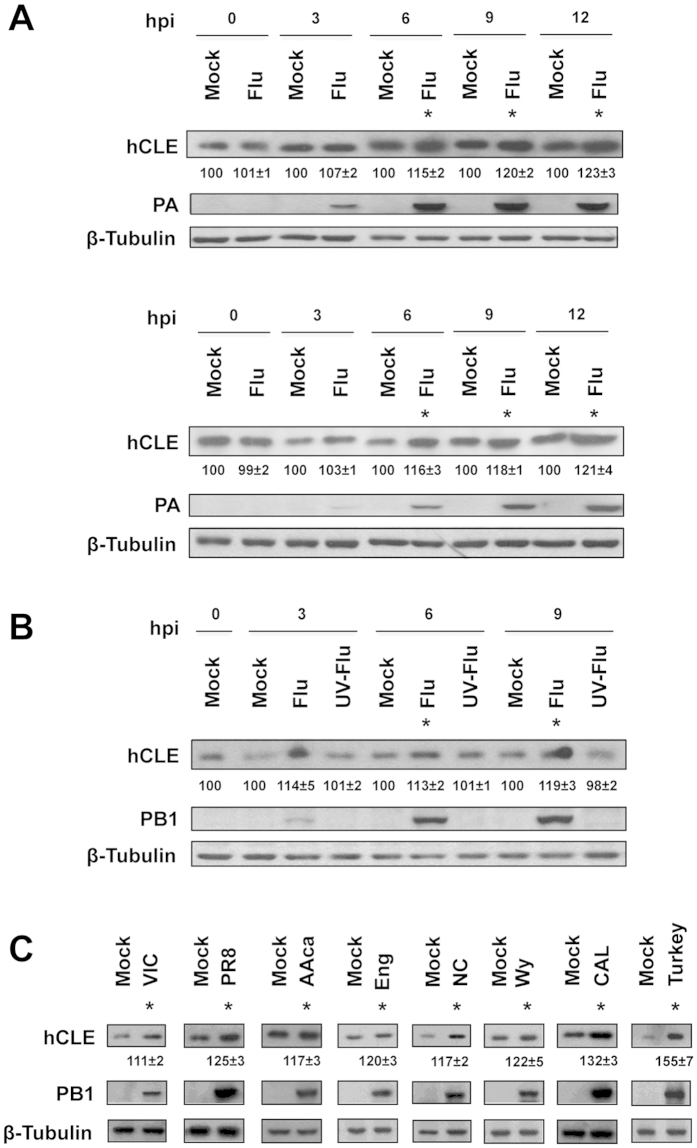
hCLE protein accumulation increases along influenza virus infection. (**A**) Cultures of A549 (top) and HEK293T cells (bottom) were infected at 3 PFU/cell with IAV WSN strain; at indicated times post-infection (hpi), hCLE was monitored in total cell extracts by Western blot. Polymerase PA subunit and β-tubulin levels were used as infection and loading controls, respectively. Panels are derived from cropped blots with lanes 1–4 belonging to 1 membrane and lanes 5–10 to another. All samples are from the same experiment; gels and membranes were loaded, run and blotted at the same time and under the same conditions. (**B**) HEK293T cells were mock-infected or infected with UV-inactivated (UV-Flu) or non-inactivated IAV virus (Flu) and levels of hCLE, PB1 and β-tubulin were monitored as above. (**C**) A549 cells were infected with different laboratory-passaged strains or natural IAV isolates. At 12 hpi, hCLE and the indicated proteins in total cell extracts were monitored by Western blot. Lanes: Mock, mock-infected cells; VIC, A/Victoria/3/75 (H3N2)-infected cells; PR8, A/PR/8/34 (H1N1)-infected cells; AAca, A/Ann Arbor/6/60 (H1N1) cold adapted-infected cells; Eng, A/England/1/51 (H1N1)-infected cells; NC, A/New Caledonia/20/99 (H1N1)-infected cells; Wy, A/Wyoming/3/2003 (H3N2)-infected cells; CAL, A/California/07/2009 (H1N1)-infected cells; Turkey, A/Turkey/Wisconsin/66 (H9N2)-infected cells. Quantification of hCLE amount during virus infection, normalised to the β-tubulin levels is shown beneath hCLE blots. An arbitrary level of 100 was assigned to each mock-infected sample and influenza-infected samples were referred to the corresponding mock-infected sample. A representative experiment is shown of three to five independent experiments. The asterisks indicate P < 0.05 (Student’s t test).

To examine hCLE behaviour during IAV infection, we infected cultured HEK293T or A549 cells with IAV WSN strain and monitored hCLE protein levels by Western blot. In contrast with the general cellular shut-off induced by IAV, hCLE accumulation increased continuously throughout the infection cycle (Fig. 1A, top (A549) and bottom (HEK293T)). To test whether viral replication is necessary for the increase in hCLE levels, IAV WSN stocks were UV-inactivated and used to infect HEK293T cells. Cell extracts were obtained at different times (hours) post-infection (hpi) and hCLE levels were determined. Intact virus infection led to increased hCLE levels (Fig. 1B, Flu lines), whereas protein levels remained unaltered when the virus was UV-inactivated (Fig. 1B, UV-Flu lines), which indicated that viral replication is needed to promote the hCLE increase. To study whether this increase constitutes a general feature of IAV infection, we infected cultures of A549 cells with various laboratory strains, vaccine donor strains and natural human isolates of the H3N2, H1N1 and H2N2 subtypes, as well as an H9N2 subtype avian strain, and monitored hCLE levels. The results showed that the increase in hCLE levels is a general effect of IAV infection (Fig. 1C).

### Specificity of influenza virus-induced hCLE accumulation

**Figure 2:**
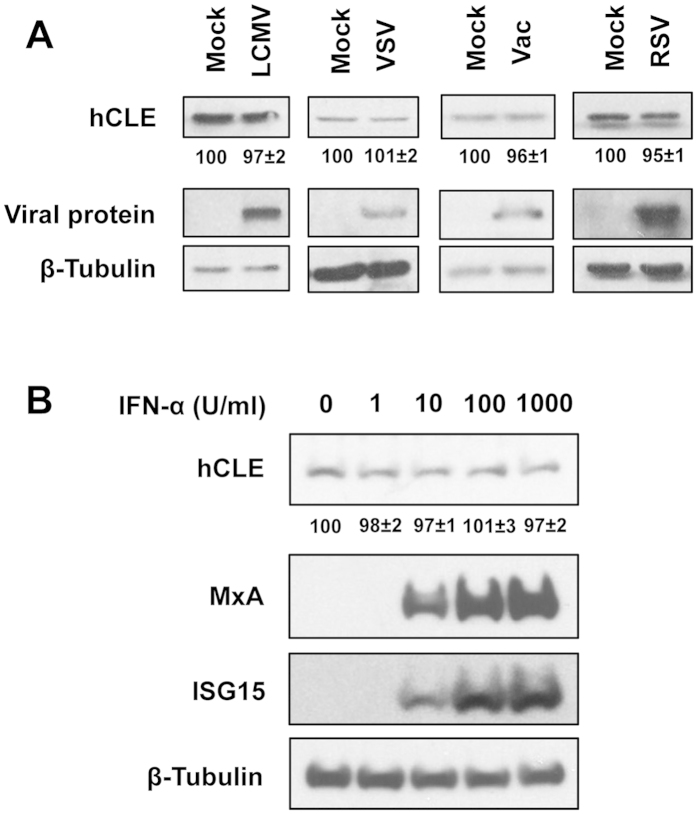
Other viruses and IFN-α treatment do not induce hCLE protein accumulation. (**A**) HEK293T cells were mock-infected or infected with lymphocytic choriomeningitis virus (LCMV), vesicular stomatitis virus (VSV), vaccinia virus (Vac) or respiratory syncytial virus (RSV), and hCLE accumulation level in total cell extracts was monitored by Western blot. Levels of LCMV NP, VSV N, vaccinia virus 39 K and RSV F proteins were used as infection controls for each virus, and β-tubulin was used as loading control. (**B**) A549 cells were treated with various doses of IFN-α for 16 h and induction of hCLE protein was determined by Western blot. MxA and ISG15 proteins were used as positive controls for IFN-α induction and β-tubulin as loading control. Quantification of hCLE amount normalised to β-tubulin levels is shown beneath the hCLE blots. An arbitrary level of 100 was assigned to each mock-infected (**A**) or untreated (**B**) sample and the virus-infected or IFN-treated samples were referred to the corresponding mock-infected or untreated sample. A representative experiment is shown of three to five independent experiments.

We next examined whether hCLE accumulation also increases after infection with other viruses using different gene expression strategies. These included segmented negative-strand RNA viruses such as lymphocytic choriomeningitis virus (LCMV), an arenavirus that carries out cap-snatching in cytoplasm^33^, the non-segmented negative-strand RNA virus vesicular stomatitis virus (VSV), which shares with IAV the activation of the RIG-I RNA sensor during infection^34^ and sensitivity to the cellular MxA protein^35^, as well as respiratory syncytial virus (RSV), which causes respiratory tract infections in humans^36^. We also evaluated vaccinia virus (Vac), a poxvirus that replicates in host cell cytoplasm. HEK293T cells were mock-infected or infected with LCMV, VSV, Vac or RSV and hCLE accumulation levels were monitored by Western blot in total cell extracts. Levels of NP (for LCMV), N (for VSV), 39 K (for Vac) and F (for RSV) proteins were used as infection controls, and β-tubulin as loading control (Fig. 2A). None of these viruses increased hCLE accumulation, suggesting that its enhancement following IAV infection is a specific feature of influenza viruses.

Type I interferons (IFN) are a large group of IFN proteins released by host cells in response to pathogens. IAV-infected cells release IFN, which alerts nearby cells and activates their anti-viral defences. IFN activities include upregulation of hundreds of IFN-stimulated genes (ISG), which have numerous antiviral functions^37^. Given this activity, we tested whether IFN treatment stimulates hCLE accumulation. A549 cells were treated with different IFN-α doses for 16 h and hCLE protein induction was determined by Western blot. hCLE accumulation levels remained unchanged after IFN-α addition, whereas we found clear dose-dependent induction of the IFN-stimulated proteins MxA and ISG15 (Fig. 2B). These data indicate that hCLE stimulation is not triggered by IFN-α, and specifically requires IAV replication.

### hCLE is associated to viral ribonucleoproteins in the cytosol of infected cells

**Figure 3:**
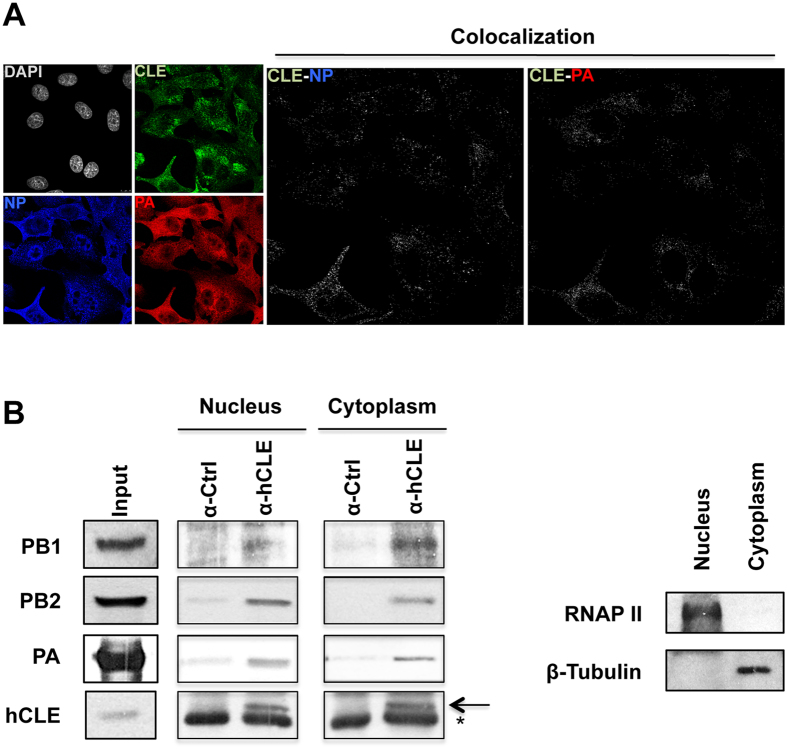
hCLE colocalises and associates with viral RNP in the cytoplasm of infected cells. (**A**) Immunofluorescence analysis using anti-hCLE, -NP and -PA antibodies. A549 cell cultures were infected with IAV WSN strain; at 9 hpi, cells were fixed and processed for immunofluorescence by confocal microscopy. Eight consecutive single confocal sections were obtained and those with maximum representation of cytosol (basal sections) were selected. CLE-NP and CLE-PA panels show the signals common to both antibodies obtained with the colocalisation mask. (**B**) HEK293T cell cultures were infected with IAV WSN strain; at 6 hpi, nuclear and cytosolic extracts were obtained for immunoprecipitation studies. hCLE, PB2, PA, and PB1 were monitored in Western blot. Input, HEK293T extracts; α-Ctrl, immunoprecipitate with control preimmune serum; α-hCLE, immunoprecipitate using a specific anti-hCLE antibody. Arrow, hCLE protein; asterisk, the immunoglobulin light chain. Right panel, nuclear and cytosolic extracts were analysed by SDS-PAGE and Western blot to confirm correct subcellular fractionation using nuclear (RNAP II) and cytosolic (β-tubulin) markers.

The analysis of hCLE intracellular distribution showed that it is a nucleo-cytoplasmic shuttling protein found both in the nucleus and in cytoplasm^22^,^26^. Previous studies showed that hCLE associates with viral polymerase and colocalises with vRNP in the nucleus, where it regulates viral RNA transcription and replication^24^. hCLE levels increased mainly at late times post-infection, which suggested a role late in IAV infection. We therefore tested whether hCLE associates with progeny vRNP exported to the cytoplasm of infected cells, using immunofluorescence (Fig. 3A) and immunoprecipitation experiments (Fig. 3B). Single basal confocal sections with maximum representation of the cytosolic compartment were used for immunofluorescence analyses of infected A549 cells. We examined colocalisation between hCLE and vRNP PA and NP proteins using specific antibodies and colocalisation masks to generate binary images that show only overlapping pixels (white spots; see Methods). The results indicated that hCLE and vRNP colocalised in infected cell cytoplasm (Fig. 3A, colocalisation panels).

To confirm hCLE-vRNP colocalisation, we performed a triple immunofluorescence assay using antibodies to hCLE, NP viral protein as a marker for vRNP, and Rab11, a recycling endosome marker that colocalises with influenza vRNP and is essential for their transport to the plasma membrane^38^,^39^,^40^. hCLE colocalised with Rab11 and with NP (Supplementary Figure 1A), which confirmed hCLE colocalisation with cytoplasmic vRNP. To evaluate the relevance of this finding, we compared hCLE colocalisation with NP and with the non-RNP viral protein HA (Supplementary Figure 1B)^40^. As predicted, there was greater hCLE colocalisation with NP protein than with HA. Next, we examined hCLE association with viral polymerase in nuclear and cytosolic fractions of the infected cells in immunoprecipitation experiments using anti-hCLE or isotype control antibodies. Correct cell fractionation was verified by Western blot with anti-RNAP II (nucleus) and β-tubulin antibodies (cytoplasm) (Fig. 3B, right). All three polymerase subunits were co-immunoprecipitated by anti-hCLE antibodies, indicating that hCLE associates with nuclear and cytoplasmic polymerase complexes (Fig. 3B, left). This result suggests that hCLE has different functions in the control of the IAV life cycle depending on its subcellular distribution.

### hCLE is incorporated into IAV particles

**Figure 4:**
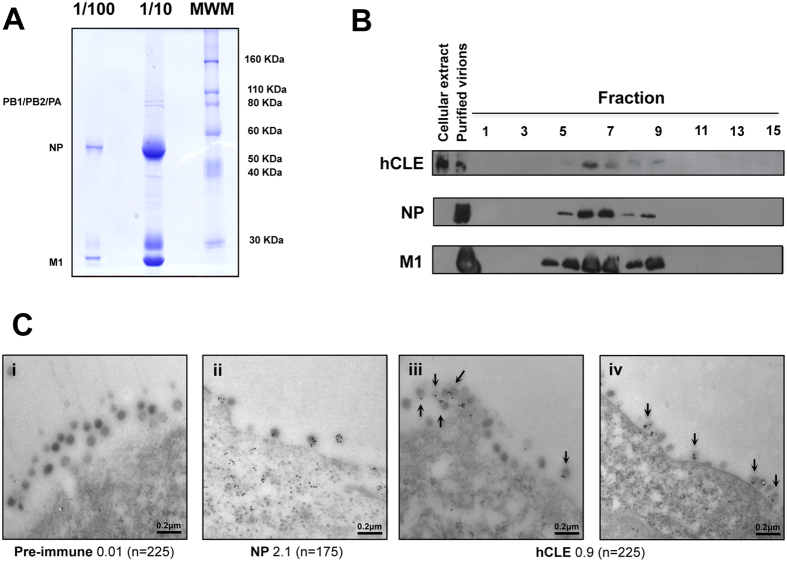
hCLE incorporation into influenza A virus particles. (**A**) Coomassie staining of purified IAV particles. IAV WSN strain was purified by sedimentation on a 33% sucrose cushion and a 33–50% sucrose step gradient. (**B**) Purified virions were further sedimented on a 30–60% linear sucrose gradient. Fractions were taken from the top and analysed by Western blot for the presence of NP, M1 and hCLE, as indicated. hCLE mobility in a total cell extract and the mobility of NP and M1 markers from purified virus are shown (left). (**C**) Immunogold labelling of hCLE in IAV virions. HEK293T cells were infected at 1 PFU/cell with IAV WSN strain. At 7 hpi, cells were fixed, and NP and hCLE were analysed in viral particles by immunogold labelling with pre-immune (i), anti-NP (ii) or anti-hCLE (iii-iv) antibodies. Labelled samples were negatively stained with uranyl acetate and visualised by electron microscopy. Arrows indicate hCLE inside IAV virions. The number of gold particles per virion is shown beneath the figure (*n* = number of virions counted).

hCLE has a nuclear role as a transcriptional activator specific for RNAP II. It also has a function in cytosol, as it has been associated to protein shuttling complexes carrying RNA^26^ and as a component of cytosolic RNA-transporting granules in neurons^41^,^42^. As hCLE associates with vRNP in the cytosol (Fig. 3) as well as with active reconstituted recombinant RNP^22^, it is possible that this interaction is maintained later in infection and that hCLE might be incorporated into virions. To analyse this possibility, we infected HEK293T cells with IAV WSN strain, and purified viral particles from the supernatants by successive sedimentation through a sucrose cushion (Fig. 4A) and a linear sucrose density gradient (Fig. 4B) (see Methods). The presence of virus particles in the gradient fractions was determined by Western blot with NP and M1 protein-specific antibodies; the potential hCLE association was also monitored by Western blot. The results showed exact co-sedimentation of viral proteins with hCLE (Fig. 4B). To confirm the presence of hCLE in IAV virions, we infected HEK293T cells with IAV WSN strain for 7 h, after which they were washed and processed for immunogold labelling and electron microscopy. Cell preparations were stained with anti-NP or -hCLE antibodies or with hCLE preimmune serum, and analysed by electron microscopy. Control preimmune serum showed only a background signal in the preparations (0.01 gold particles/virion) (Fig. 4C, panel i), whereas we found clear NP (2.1 gold particles/virion) (Fig. 4C, panel ii) and hCLE signals (0.9 gold particles/virion) (Fig. 4C, panels iii-iv).

**Figure 5:**
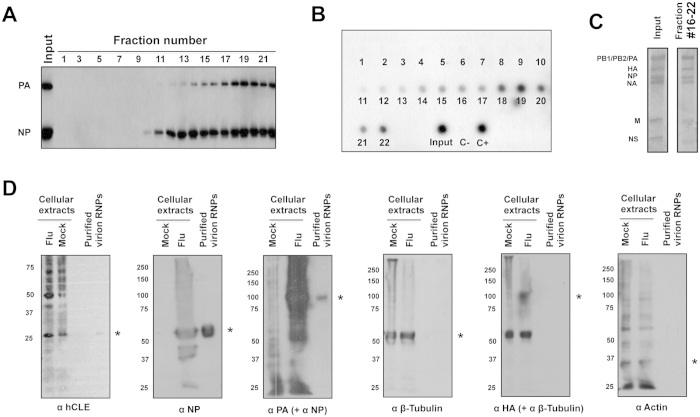
hCLE associates with vRNP inside influenza virions. IAV WSN strain was purified over a 33% sucrose cushion, viral particles disrupted by detergent treatment and virion RNP fractionated over a 33–70% glycerol gradient. Fractions were analysed by Western blot using anti-PA and -NP antibodies (**A**) and *in vitro* transcription using ApG as primer (**B**). The activity of the input sample applied to the gradient, as well as positive and negative controls (C+ and C), is indicated (bottom). Fractions 16–22 were selected and pooled. Virion RNAs were purified and analysed by denaturing polyacrylamide-urea gel electrophoresis and silver staining (**C**). The positions of the eight viral RNA segments are indicated (left). hCLE, the cellular proteins β-Tubulin and Actin, the non RNP virion protein HA and the indicated viral RNP proteins in these mixed fractions was evaluated by Western blot (**D**). Mock- or influenza-infected cell extract were used as control. Asterisks indicate the corresponding protein.

To verify the specificity of hCLE association with virus particles, virions were purified from supernatants of MDCK-infected cells, the purified viral particles were disrupted by detergent treatment, and the resulting vRNP were purified by ultracentrifugation in a glycerol gradient. Western blot and *in vitro* transcription assays were used to test each fraction for PA and NP proteins as well as for polymerase activity (Fig. 5A,B). Fractions 16–22 were enriched in active vRNP. These fractions were combined and RNA content examined, which showed all viral RNA segments, whereas no cellular RNA was detected (Fig. 5C). The presence of hCLE in these pooled fractions of purified vRNP was tested by Western blot (Fig. 5D). In addition to vRNP proteins (PA and NP), hCLE, was detected in the preparation, whereas we were unable to identify a non RNP virion protein (HA) as well as cellular proteins known to interact with the RNP and also be incorporated into influenza viral particles (Actin and β-Tubulin)^43^,^44^,^45^. These data indicates a close hCLE association to the vRNP packaged in virions.

## Discussion

Recent reports identify a wide range of cell factors that affect the IAV life cycle. These factors participate in cellular processes such as transcription, splicing and antiviral response, and should have effects on different stages of the IAV life cycle, such as genome transcription and replication, nuclear import and export of vRNP, translation, or virus morphogenesis and budding^46^,^47^,^48^,^49^,^50^,^51^. One of these factors is hCLE, which associates with the PA subunit^22^; it was later confirmed as a host factor that interacts with the viral polymerase complex^24^,^52^ and its knockdown decreases IAV RNA transcription and replication as well as production of infectious virus^24^.

Although the cellular functions of hCLE are not fully understood, data indicate an important role in cellular mRNA transcription, since its knockdown inhibits mRNA synthesis by 50%^25^. Several proteomic analyses describe hCLE as an important component of nuclear complexes that are involved in transcription modulation such as the human spliceosome^53^, the 7SK snRNA methylphosphate capping complex^54^ and the tRNA-splicing ligase complex^55^. Proteomic analysis of hCLE reported that in addition to its association to RNAP II^25^, it interacts with nuclear and cytosolic proteins involved in RNA transport^26^ and other functions. hCLE shuttles between the nucleus and the cytoplasm, but its nuclear import requires active transcription^26^. These data and the presence of RNA in nuclear and cytoplasmic hCLE complexes^26^ suggest that it could be involved in the nucleo-cytoplasmic transport of newly synthesised RNAs. Here we show that hCLE interacts not only with the viral polymerase in the nucleus, but also associates with progeny vRNP in cytoplasm (Fig. 3). This suggests hCLE involvement in viral transcription and replication processes in the nucleus as well as in accompanying progeny vRNP during their export to the cytoplasm. A recent report confirmed hCLE interaction with the vRNP components PB2, PA and NP and described additional interactions with NA and M1, all virion protein components^50^. This close interaction between hCLE and vRNP supports our finding that hCLE is incorporated into IAV virions, and is tightly bound to the viral RNP (Figs 4 and 5).

Proteomic analysis of IAV virions reported 36 host proteins within the viral particles^56^, a large fraction of which are abundant cytosolic proteins. Such proteins might be packaged non-specifically, as they were found in virions of quite diverse virus families such as herpes-, pox- or retroviruses^57^,^58^, whereas others might have a role in viral replication. A recent tandem mass spectrometry study of IAV virion composition described several hundreds of incorporated host proteins, a large number present at low abundance and a smaller set of major virion components^49^. Virions incorporated ubiquitin, annexins, cytoskeletal proteins and glycolytic enzymes, as well as cyclophilin A, small GTPases and other signalling regulators^49^. Neither of these studies identified hCLE as an influenza virion component; although the host factors incorporated into the IAV virions belong to different functional categories, none is a transcription-related factor^56^ or is particularly relevant^49^. There are few reports of transcription factor incorporation into viral particles. Among these, the RNA helicase DDX3, which is involved in pre-mRNA splicing and RNA transport, was found in hepatitis B virus nucleocapsids^59^; SAP18, a component of the histone deacetylase complex, is packaged in HIV-1 virions^60^, and HIC2, a transcriptional repressor, was detected in Moloney murine leukaemia purified vector preparations^61^. Here we show that hCLE, a positive modulator of RNAP II, is incorporated into IAV virions.

Host proteins specifically packaged in viral particles are likely to interact with either a viral protein or the viral genome. Such proteins would probably be involved in the virus replication cycle, either at late stages during virus assembly or at early stages of entry into the new target cell. The reason for hCLE incorporation into viral particles remains unclear, although several hypotheses can be proposed based on data regarding its protein interactions and functions. hCLE is involved in the traffic of RNA-containing granules^38^,^39^ and interacts with the PA polymerase subunit in two regions (amino acids 493–512 and 557–574)^22^. IAV PA has well-characterised functions during infection, as a cap-dependent endonuclease^11^,^12^ and the ability to induce proteolysis^62^,^63^,^64^; in addition, PA mutations on residues 507 and 508 cause modest perturbation of RNA expression, but completely eliminate formation of plaque-forming virus^65^. By virtue of the viral polymerase binding to vRNA, hCLE might collaborate with other cell proteins that direct intracellular vRNP trafficking toward viral assembly. The PA residues whose mutations abolish virus formation map within the first of the two PA-hCLE binding regions. These data suggest that impaired PA-hCLE interaction may be responsible for the PA phenotype. On the other hand, hCLE interacts with two polymerases that are closely associated during the IAV infection cycle, cellular RNAP II and the viral polymerase, and positively modulates both activities. hCLE could thus facilitate viral and cellular polymerase interaction for the productive cap-snatching necessary for viral transcription.

CLE/C14orf166 is present in many organisms, and database searches show considerable sequence conservation among humans, swine, mice, and fowl (Supplementary Figure 2), indicating a preserved function for this protein. These observations suggest a general role of CLE in IAV replication and make CLE a potential candidate for the design of new broad-spectrum antiviral compounds.

## Materials and Methods

### Biological materials

Cell lines used in this study were MDCK (canine kidney) and HEK293T and A549 (human kidney and respiratory epithelium). The influenza virus strains A/Victoria/3/75 (H3N2), A/WSN/33 (H1N1), A/PR/8/34 (H1N1), A/New Caledonia/20/99 (H1N1), A/England/1/51 (H1N1), A/Wyoming/3/2003 (H3N2), A/Ann Arbor/6/60 cold-adapted (H2N2), A/California/07/2009 (H1N1) and A/Turkey/Wisconsin/66 (H9N2) were propagated and titrated in MDCK cells. Recombinant vaccinia virus vTF7-3 was provided by B. Moss, lymphocytic choriomeningitis virus was a gift of J.C. de la Torre, vesicular stomatitis virus (New Jersey serotype) was supplied by M. Esteban, and respiratory syncytial virus was kindly provided by I. Martínez. Recombinant Universal type I interferon was purchased from PBL Assay Science (Piscataway, NJ).

### Virus infection

Cells were infected with IAV at the multiplicity of infection (MOI) specified in each experiment. After 1 h, cells were washed with PBS and overlaid with DMEM growth medium (Dulbecco’s minimal essential medium). Cells were infected with RSV (MOI of 5), VSV (MOI of 3), Vac (MOI of 5) or LCMV (MOI of 3) and total cell extracts were harvested in Laemmli sample buffer after 24, 8, 9 and 10 h, respectively.

### Immunoprecipitation

Immunoprecipitation studies were performed as described^24^. Briefly, HEK293T cells were infected with WSN IAV strain (3 PFU/cell). At 6 hpi, cells were collected in PBS and pelleted by centrifugation, resuspended in a solution containing 10 mM Tris-HCl pH 8.0, 10 mM KCl, 0.1% NP-40, 1 mM EDTA, 1 mM dithiothreitol (DTT) and protease inhibitors (Complete, Roche), incubated on ice (5 min), and centrifuged (3000 rpm, 5 min, 4 °C). The supernatant was recovered and supplemented with NaCl to a final concentration of 150 mM (cytosolic extract). The sedimented nuclei were extracted in a solution of 20 mM Tris-HCl pH 8.0, 0.4 M NaCl, 10% glycerol, 1.5 mM MgCl_2_, 1 mM EDTA, 1 mM DTT and protease inhibitors (Complete) for 30 min on ice with occasional vortexing, and then centrifuged (15000 rpm 4 °C, 15 min). The supernatant (nuclear extract) was adjusted to a final concentration of 150 mM NaCl, 10 mM Tris-HCl pH 8.0 and 0.1%. NP-40. Nuclear or cytosolic extracts were immunoprecipitated with a rabbit anti-hCLE or a pre-immune antibody^22^. Immune complexes were washed 10 times with a buffer containing 100 mM NaCl, 10 mM Tris-HCl (pH 8.0), 0.5% Triton X-100, 0.5 mM DTT and 0.2 mM EDTA, and the immunoprecipitated proteins analysed by Western blot.

### Western blotting

Western blotting was carried out as described^27^ using the following primary antibodies: rabbit polyclonal anti-hCLE (1:1000, Abcam), monoclonal antibody (mAb) for anti-RNAP II (8WG16; 1:500, Covance), mAb anti-PA (antibodies 2 and 9; both at 1:200)^66^, rabbit polyclonal anti-PB1 (1:1000)^67^, anti-PB2 mAB (antibodies 8 and 28; both at 1:100)^68^, rabbit polyclonal anti-NP protein (1:5000)^69^, mouse anti-M1 protein mAb (1:200)^70^ and mouse anti-β-tubulin mAb (1:15,000; Sigma). Anti-vaccinia virus 39 K and VSV N proteins were kindly provided by D. Rodríguez (CNB-CSIC, Madrid, Spain), anti-LCMV NP and RSV F protein antibodies were gifts of J.C. de la Torre (The Scripps Research Institute, La Jolla, CA) and I. Martinez (ISCIII, Madrid, Spain), respectively. Antibodies to MxA and ISG15 were generously provided by U. Garaigorta (The Scripps Research Institute).

### Immunofluorescence

Cultured A549 cells were infected with the IAV WSN strain (MOI of 5 PFU/cell). At 9 hpi, cells were fixed with 3.7% formalin (20 min, room temperature) and stored in PBS. For immunofluorescence, cells were permeabilised in PBS containing 0.5% Triton X-100 (5 min) and incubated with the following primary antibodies diluted in PBS/0.1% BSA (w/v): rabbit anti-hCLE (1:1000)^22^, rat polyclonal anti-NP (1:2000)^27^, monoclonal anti-PA (1:2)^66^, monoclonal anti-Rab11 (BD Biosciences), and monoclonal anti-influenza HA antibody (1:3) generously provided by J.A. Melero (ISCIII, Madrid, Spain). Confocal microscopy was performed with a Leica TCS SP5 laser scanning system. Images of 1024 × 1024 pixels and an eight bit gray scale depth were acquired sequentially every 0.2–0.3 μm using LAS AF version 2.2.1 software (Leica) and analysed using LAS AF and MetaMorph Premier version 7.5.2 image analysis software (Molecular Devices). For colocalisation analyses, single confocal sections and the colocalisation mask that produces binary images showing only overlapping pixels (white spots) were used.

### Immunogold labelling and electron microscopy

HEK293T cells were infected at 1 PFU/cell with the WSN strain. At 7 hpi, cells were fixed in cold PBS containing 4% paraformaldehyde and 0.1% glutaraldehyde (30 min, 4 °C). The cell layer was carefully removed and centrifuged (1.500 rpm, 5 min, 4 °C). Fixation buffer was removed and cells were resuspended in PBS. Cells were cryoprotected by progressive inclusion of up to 30% glycerol and cryofixed by plunge-freezing in liquid ethanol at −180 °C in a Leica CPC cryopreparation chamber. Cryosubstitution was carried out by incubating cells in methanol containing 0.5% uranyl acetate (50 h, −90 °C) in a Leica AFS freeze substitution chamber. Samples were then embedded in Lowicryl HM20 resin (20 h, −40 °C) and polymerised under UV light (48 h, −40 °C and 48 h, 20 °C). Ultrathin sections (60–70 nm) were obtained in a Leica–EM-UC6 ultramicrotome and collected on formvar-coated gold grids. Cell sections were blocked in TBS containing 0.2% Tween-20 and 1.5% BSA (15 min, room temperature), then incubated with pre-immune (1:20), anti-hCLE^22^ (1:20), or anti-NP^69^ antibodies(1:500; 1 h, room temperature). After washing in 0.2% Tween 20-TBS, grids were incubated with a 10 nm colloidal gold-conjugated goat anti-rabbit antibody (1:40; 45 min), washed in 0.2% Tween 20-TBS and water, dried and contrasted with saturated uranyl acetate (20 min, room temperature). Samples were analysed on a Jeol-JEM 1010 transmission electron microscope at 100  KV and images captured on a Gatan Erlangshen ES 1000 W camera.

### Virion purification

Virions were purified as described^71^. Briefly, cells were infected at low MOI (10^−3 ^PFU/ml). Culture supernatants were pre-cleared by centrifugation (10,000 rpm, 10 min, 4 °C) and the supernatant was centrifuged on a 33% sucrose cushion (26,000 rpm, 2.5 h, 4 °C) in a SW28 rotor. The pellet was resuspended in TNE (100 mM NaCl, 5 mM EDTA, 50 mM HCl-Tris pH 7.5) and centrifuged over a 33–50% sucrose-TNE step gradient (40,00 rpm, 1 h, 4 °C) in a SW41 rotor. The 33–50% interface was collected and diluted in the same buffer. For gradient fractionation, the virus pellet was centrifuged over a continuous 30–60% sucrose gradient. Laemmli buffer was added to the resulting fractions and samples were loaded onto polyacrylamide gels for Western blot analysis.

### Virion RNP purification, RNA analysis and *in vitro* transcription

RNP from IAV particles were purified as described^72^. Supernatants of infected MDCK cells were collected 40 hpi, pre-cleared by centrifugation (9,000 rpm, 15 min, 4 °C). The supernatant was centrifuged (25,000 rpm, 2.5 h) in a SW28 rotor. The viral pellet was diluted in TNE and centrifuged on a 33% sucrose cushion (40,000 rpm, 1 h, 4 °C) in a SW41Ti rotor. The final pellets were lysed (45 min, 30 °C) in a buffer containing 100 mM Tris-HCl, 100 mM NaCl, 5 mM MgCl_2_, 1% NP40, 3 mM DTT and 10 mg/ml lysolecithin pH 7.5. The extract was centrifuged on a 33% to 70% glycerol gradient in TN buffer (150 mM NaCl, 50 mM Tris-HCl, pH 7.8) (45,000 rpm, 4 h, 4 °C) in a SW55Ti rotor. The RNA of purified RNP was isolated as described^69^. RNA was ethanol precipitated, resuspended in formamide loading buffer and analysed by electrophoresis in a 4% polyacrylamide-urea denaturing gel. The activity of purified virion RNP was tested by ApG-primed *in vitro* transcription^69^. Samples were incubated in a buffer containing 50 mM Tris-HCl, 5 mM MgCl_2_, 100 mM KCl, 1 mM DTT, 10 μ/ml actinomycin D, 1 U/μl RNAsin, 1 mM ATP, 1 mM CTP, 1 mM UTP, 10 μM α-^32^P-GTP (20 μCi/μmol) and 100 μM ApG (1 h, 30 °C). The RNA synthesised was TCA precipitated, filtered through a nylon filter in a dot-blot apparatus and quantified in a phosphorimager.

## Additional Information

**How to cite this article**: Ariel, R.-F. *et al*. hCLE/C14orf166, a cellular protein required for viral replication, is incorporated into influenza virus particles. *Sci. Rep*. **6**, 20744; doi: 10.1038/srep20744 (2016).

## Supplementary Material

Supplementary Information
