# Supplementary material for: hCLE/C14orf166, a cellular protein required for viral replication, is incorporated into influenza virus particles
Source: Sci Rep. 2016 Feb 11;6:20744. doi: 10.1038/srep20744 (PMC4749964; doi:10.1038/srep20744)
Supplement: Supplementary Information [file srep20744-s1.pdf]

hCLE/C14orf166, a cellular protein required for viral replication, is incorporated into influenza virus particles

Ariel Rodriguez-Frandsen[1,2§,*], Susana de Lucas[1,2,¶], Alicia Pérez-González[1,2,†], Maite Pérez-Cidoncha[1,2,#], Alejandro Roldan-Gomendio[1,£], Alejandra Pazo[1,2], Laura Marcos-Villar[1,2], Sara Landeras-Bueno[1,2], Juan Ortín[1,2] and Amelia Nieto[1,2*]

A

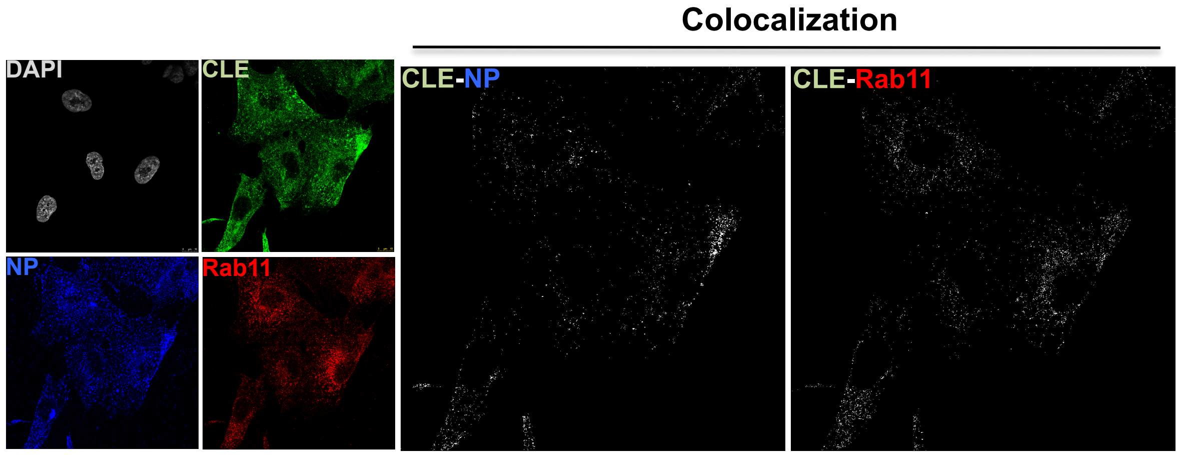

B

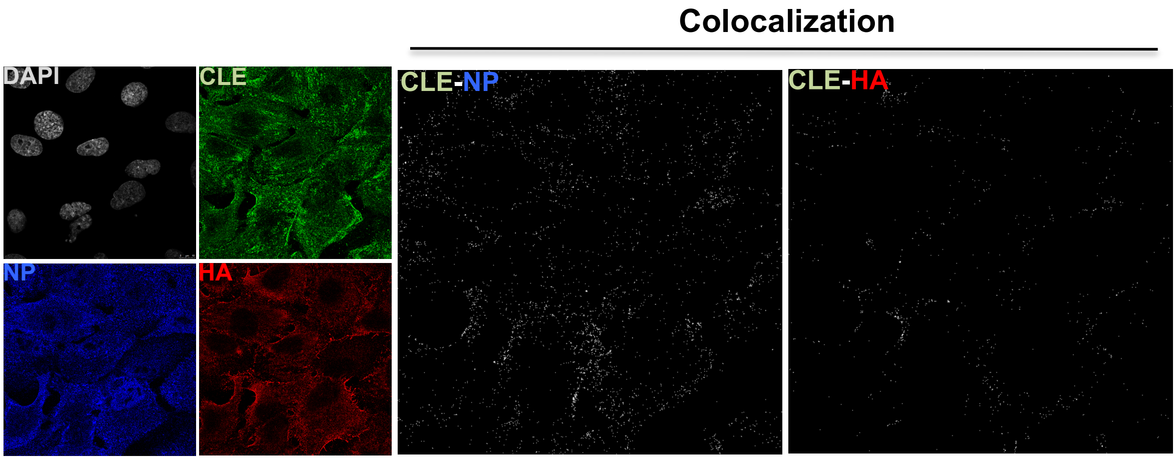

Supplemental Figure 1: Colocalization of hCLE with the endosomal marker Rab11 and the influenza virus HA proteins. Cultures of A549 cells were infected with IAV WSN strain. At 9 hpi the cells were fixed and processed for immunofluorescence by confocal microscopy. Eight consecutive single confocal sections were obtained and those with higher representation of the cytosol (basal sections) were selected. (A) Immunofluorescence analysis using antibodies against hCLE, NP and the recycling endosomal marker Rab11. The hCLE-NP and hCLE-Rab11 panels show the signals common to both antibodies obtained with the colocalization mask, respectively. (B) Immunofluorescence analysis using antibodies against hCLE, NP and HA. The hCLE-NP and hCLE-HA panels show the signals common to both antibodies obtained with the colocalization mask, respectively.

```
Homo sapiens       ------------------------------------------------------------
Sus scrofa       1 MAIAESKYTVLCGVYQHREENNSVDVPQRGPASPTGATIASRLPTPSRRVVGACAARSSS
Mus musculus       ------------------------------------------------------------
Gallus gallus      ------------------------------------------------------------

Homo sapiens       -----------------MFRRKLTALDYHNPAGFNCKDETEFRNFIVWLEDQKIRHYKIE
Sus scrofa         VLPSRLKTGDRSEQTGTMFRRKLTALDYHNPAGFNCKDETEFRNFIVWLEDQKIRHYKIE
Mus musculus       -----------------MFRRKLTALDYHNPSGFNCKDETEFRNFIVWLEDQKIRHYKIE
Gallus gallus      -----------------MFRRKLSALDYHNPGGFNCRDETEFRNFIVWLEDQKIRHYKIE
                                    ******:*******:****:***********************

Homo sapiens       DRGNLRNIHSSDWPKFFEKYLRDVNCPFKIQDRQEAIDWLLGLAVRLEYGDNA-------
Sus scrofa         DRGNLRNIHSSDWPKFFEKYLRDVNCPFKIQDRQEAIDWLLGLAVRLEYGDNANHISTTA
Mus musculus       DRGNLRNIHSSDWPKFFEKYLKDVNCPFKIQDRQEAIDWLLGLAVRLEYGDNA-------
Gallus gallus      DRGNLRNIHSDDWPKSIEKYMKDVNCPFKMQERQETVDWLLGLAVRLEYGDNA-------
                   **********:**** :***::*******:*:***::****************

Homo sapiens       ----------EKYKDLVPDNSKTADNATKNAEPLINLDVNNPDFKAGVMALANLLQIQRH
Sus scrofa         KDGILGTKFTEKYKDLVPDNTKNADNAAKNAEPLINLDVNNPDFKAGVMALANLLQIQRH
Mus musculus       ----------EKYKDLVPDNRKNTDNAAKNAEPLINLDVNNPDFKAGVMALANLLQIQRH
Gallus gallus      ----------DKYKDSTPDGAKNTDNTAKNAEPLINLDVNNPDFKAGVMALANLLQIQRH
                             :**** :**: *::**::********************************

Homo sapiens       DDYLVMLKAIRILVQERLTQDAVAKANQTKEGLPVALDKHILGFDTGDAVLNEAAQILRL
Sus scrofa         DDYLVMLKAIRILVQERLTQDAVAKANQTKEGLPVALDKHILGFDTGDAVLNEAAQILRL
Mus musculus       DDYLVMLKAIRILVQERLTQDAVAKANQTKEGLPVALEKHILGFDTGDAVLNEAAQILRL
Gallus gallus      DDYLVMLKAIREKVQD-----AIAKANQSKEGLPVALEKHILGFDTGDAVINEAAQILRL
                   ***********  **:     *:*****:********:************:*********

Homo sapiens       LHIEELRELQTKINEAIVAVQAIIADPKTDHRLGKVGR 244
Sus scrofa         LHIEELRELQTKINEAIVAVQAIIADPKTDHRLGKVGR 338
Mus musculus       LHIEELRELQTKINEAIVAVQAIIADPKTDHRLGKVGR 244
Gallus gallus      LHIEELRELQTKINEAIVAVQAIIADPKTDHRLGKVGR 239
                   **************************************
```

Supplemental Figure 2: Similarity between CLE proteins from different organisms.

A comparison of sequences of the human CLE protein (*Homo sapiens*) and its homologues from mice (*Mus musculus*), swine (*Sus scrofa*) and avian (*Gallus gallus*) is shown. (*) indicates identical and (:) similar residues.
